# Enhanced Spike-specific, but attenuated Nucleocapsid-specific T cell responses upon SARS-CoV-2 breakthrough versus non-breakthrough infections

**DOI:** 10.3389/fimmu.2022.1026473

**Published:** 2022-12-13

**Authors:** Mohamed Ibraheem Mahmoud Ahmed, Paulina Diepers, Christian Janke, Michael Plank, Tabea M. Eser, Raquel Rubio-Acero, Anna Fuchs, Olga Baranov, Noemi Castelletti, Inge Kroidl, Laura Olbrich, Bernadette Bauer, Danni Wang, Martina Prelog, Johannes G. Liese, Christina Reinkemeyer, Michael Hoelscher, Philipp Steininger, Klaus Überla, Andreas Wieser, Christof Geldmacher

**Affiliations:** ^1^ Division of Infectious Diseases and Tropical Medicine, University Hospital, LMU Munich, Munich, Germany; ^2^ German Centre for Infection Research (DZIF), Munich, Germany; ^3^ Oxford Vaccine Group, Department of Paediatrics, NIHR Oxford Biomedical Research Centre, University of Oxford, Oxford, United Kingdom; ^4^ Pediatric Rheumatology/Special Immunology, Department of Pediatrics, University Hospital Würzburg, Würzburg, Germany; ^5^ Department of Pediatrics, University Hospital Würzburg, Würzburg, Germany; ^6^ Institute of Clinical and Molecular Virology, University Hospital Erlangen, Friedrich-Alexander-Universität Erlangen-Nürnberg, Erlangen, Germany

**Keywords:** COVID-19, adaptive immunity, vaccine, SARS Cov 2, breakthrough infection, T cell response

## Abstract

SARS-CoV-2 vaccine breakthrough infections frequently occurred even before the emergence of Omicron variants. Yet, relatively little is known about the impact of vaccination on SARS-CoV-2-specific T cell and antibody response dynamics upon breakthrough infection. We have therefore studied the dynamics of CD4 and CD8 T cells targeting the vaccine-encoded Spike and the non-encoded Nucleocapsid antigens during breakthrough infections (BTI, n=24) and in unvaccinated control infections (non-BTI, n=30). Subjects with vaccine breakthrough infection had significantly higher CD4 and CD8 T cell responses targeting the vaccine-encoded Spike during the first and third/fourth week after PCR diagnosis compared to non-vaccinated controls, respectively. In contrast, CD4 T cells targeting the non-vaccine encoded Nucleocapsid antigen were of significantly lower magnitude in BTI as compared to non-BTI. Hence, previous vaccination was linked to enhanced T cell responses targeting the vaccine-encoded Spike antigen, while responses against the non-vaccine encoded Nucleocapsid antigen were significantly attenuated.

## Introduction

Vaccine breakthrough infections (BTI) account for a significant portion of new COVID-19 cases (1–4). In the pre-Omicron era, SARS-CoV-2 vaccination conferred significant protection from symptomatic infection (5–7) and from severe disease upon breakthrough infection; BTI cases showed substantially lower rates of hospitalization in all age groups (4) and even when hospitalized, previous vaccination reduced morbidity and mortality of COVID-19 (8). BTI cases however are still characterized by surprisingly high upper airway viral loads during acute infection, reaching similar peak levels compared to unvaccinated individuals (1, 9, 10). The vaccination status also affects the resulting SARS-CoV-2-specific adaptive immune response; non-human primate data suggest that vaccine-induced pre-existing Spike (S)-specific antibodies and CD8 T cells play an instrumental role in the reduction of virus replication and dissemination upon BTI into tissues other than the upper respiratory tract. This is probably due to the rapid induction of anamnestic cellular responses to the vaccine-encoded S (11, 12). Clinical studies show high S-specific binding antibody titers and superior neutralization capabilities after BTI, as compared to mere vaccination (13, 14). Although several studies have investigated S-specific T cell responses on recovered COVID-19 patients versus vaccinated individuals (15, 16), few have dissected the early dynamics of SARS-CoV-2-specific T cell and antibody response during BTI in detail (17). We hypothesized that the interplay between pre-existing S-specific immunity to SARS-CoV-2 and virus dynamics during BTI impact on the adaptive immune responses with divergent dynamics between vaccine-encoded and non-encoded antigens. We therefore studied and compared T cell and antibody response dynamics against the vaccine-encoded S-protein and those targeting the non-encoded immunodominant virion Nucleocapsid (N) protein.

## Methods

### Study population

The participants were recruited from the CoVaKo (Corona Vaccine Consortium) breakthrough infection study, which is a multicentre prospective cohort study including six different university hospital centres in Bavaria, Germany, recruited from May to December 2021. Outpatient BTI (N = 24) and non-BTI (N = 15) from Munich, Germany, were enrolled within 13 days of a PCR confirmed SARS-CoV-2 infection. Weekly blood samples were collected during four visits after confirmed diagnosis. In addition, data from outpatient non-BTI cases (n = 15) matched for time after PCR diagnosis, age, and sex from a similarly designed prospective COVID-19 Cohort Munich (KoCo19) sub-study were included in the analyses; a detailed description of the study design, setting, and population was previously published (18, 19). In brief, individuals with a documented positive SARS-CoV-2 RT-PCR result were recruited in a prospective longitudinal cohort from May to December 2020 under the umbrella of the KoCo19 studies. For both studies, participants consented and were recruited as fast as possible upon RT-PCR confirmation of SARS-CoV-2 infection and followed during four weekly visits. All included patients had mild to moderate COVID-19 symptoms and had not been previously infected with SARS-CoV-2. Both cohort studies were approved by the institutional review board of Ludwig Maximilian University of Munich, Germany.

### Characterisation of SARS-CoV-2-specific T cells by intracellular staining analyses

40 ml of heparinized fresh whole venous blood was obtained during each visit. Peripheral blood mononuclear cell (PBMC) isolation using Ficoll-Paque™ Plus medium was performed within 6 hours of blood draw. The cells were washed three times and stimulated overnight 16 to 18 hours at 37°C and 5% CO_2_, in the presence of Brefeldin A (BFA, final concentration 5 μg/ml, Sigma) and the costimulatory antibodies anti-CD49d (L25, BD) and anti-CD28 (L293, BD), using three different SARS-CoV-2 specific antigens; Spike protein, PepTivator SARS-CoV-2 Prot_S (1μg/ml/peptide, Miltenyi Biotec) and the Nucleocapsid protein (1μg/ml/peptide, Miltinyi Biotec), Staphylococcal enterotoxin B (0.6 μg/ml/peptide, Sigma-Aldrich) as a positive control, and no peptide for the negative control. Following incubation, cells were stained for 20 minutes with anti-CD8 APC-A750 (clone B9.11, Beckmann Coulter) and anti-CD4 ECD (clone SFCI12T4D11, Beckmann Coulter). Cells were fixed and permeabilized using Foxp3 Fixation/Permeabilization concentrate and diluent (eBioscience), and then stained intracellularly for 30 minutes using anti-IFNy FITC (clone B27, BD Biolegend) and anti-CD3 APC-A700 (clone UCHT1, Beckmann Coulter). Samples were acquired on a CytoFlex Flow cytometer (Beckman Coulter). Gating analyses were performed using FlowJo™_V10 software (BD Life Sciences). Background subtraction was performed by subtracting IFNy+ T cell frequencies in the negative control from those in the antigen stimulated sample using Python 3.8.10.

### Assessment of SARS-CoV-2-specific binding antibody responses

Serological assays to test for SARS-CoV-2-specific binding antibodies were performed as previously published (20, 21). EDTA plasma was used to quantify binding antibodies specific for S and N protein using Roche Elecsys anti-Nucleocapsid (Ro-N-Ig) and anti-Spike-Receptor binding Domain (Ro-RBD-Ig) (both Roche, Mannheim, Germany). All assays were performed according to manufacturer’s instructions. A value above 1 unit/ml on the Ro-RBD-Ig was considered a positive antibody response towards the SARS-CoV-2 Spike response and a value above 0.8 counts on the Ro-N-Ig was considered as a positive response towards the SARS-CoV-2 Nucleocapsid.

### Statistics

The non-parametric Mann-Whitney test (python version 3.8 using package scipy, version 1.7.2) was used to compare independent continuous variables to determine the significance. A p-value ≤0.05 was considered statistically significant. Some of the conditions were compared based on area under the curve (AUC) normalised per day. The value was calculated according to the formula Σ_d_ *x_jd_*/D;*d* ∈{*d*_1_,…,*D*}with *j* indicating the patient, *d* being the day of the visit and *D* the day of the last visit. In summary, the magnitude of all measured responses was summed and divided by the number of days since PCR diagnosis at the last available visit.

## Results

### Characteristics of breakthrough and non-breakthrough infections

Blood was obtained from outpatient breakthrough infections (BTI, n= 24) and non-BTI (n= 30) during and shortly after the acute phase of SARS-CoV-2 infection (**Table 1**). Most subjects with BTI had received two vaccinations with the BNT162b2 vaccine (n = 19, BioNTech/Pfizer). 2 subjects had received 2 doses of mRNA-1273 (Moderna), 2 subjects had received 2 doses of heterologous vaccines (n=2) and one subject had received 2 doses of AZD1222 (n=1, AstraZeneca). BTI occurred with a median of 83 days (Range: 22 – 211 days) after the second vaccination. BTI and non-BTI were recruited within a median of 8 days and 3.5 days after PCR-confirmed diagnosis, respectively. The median age was 42.5 and 39.5 years in BTI and non-BTI cases, respectively. Overall, 61% of those infected were female. 37% (20/54) of the infected cases whose variants were determined were infected with the delta-variant, while 3 subjects were infected with the alpha variant. Of note, 15 non-BTI cases were recruited before the emergence of variant of concerns and hence were likely caused by the original Wuhan strain.

**Table 1:** Basic characteristics of the study population.

	Vaccine Breakthrough infection (n = 24)	Non-Breakthrough infection (n = 30)
Gender, %		
Male Female	42% (10/24)	37% (11/30)
	58% (14/24)	63% (19/30)
Median Age, y (Range)	42.5 (20 – 66)	39.5 (18 – 72)
Days after PCR+ (Range)	8 (2 - 13)	3.5 (0 - 13)
SARS-CoV-2 variant (w/δ/α/u) ^a^	0/14/3/7	0/6/0/24
Vaccine type (BB/MM/BM/AA/AB) ^b^	19/2/1/1/1	-^c^
Median days from last vaccination to PCR diagnosis (Range)	83 (22 - 211)	-^c^

a w, wild type; δ, delta stain; α, alpha strain; u, unknown strain.

b B = BNT162b2, M = mRNA-1273, A = AZD1222.

c non-BTI were not vaccinated.

### Early dynamics of T cell responses targeting the vaccine-encoded Spike and the non-encoded Nucleocapsid

**Figure 1:**
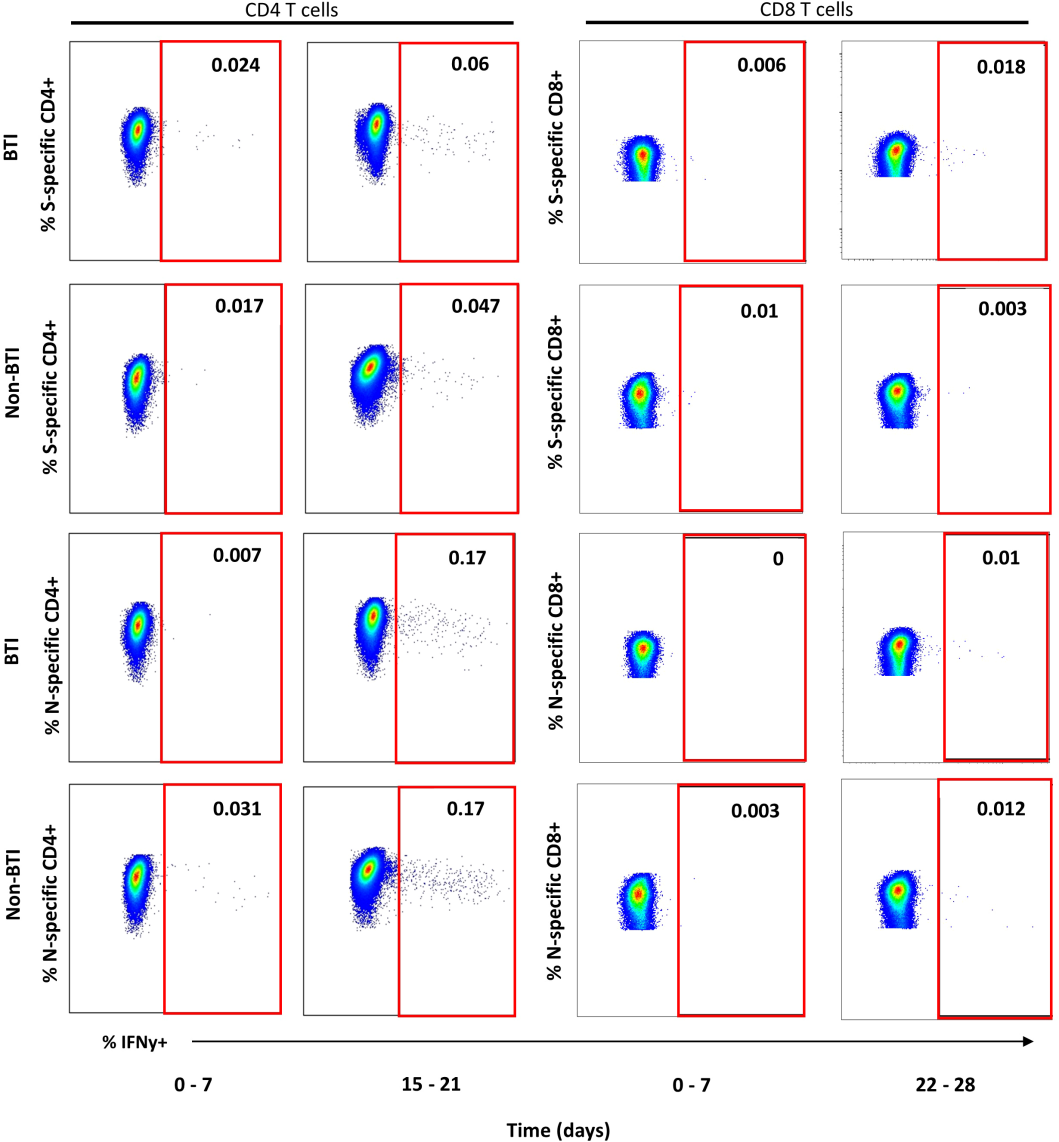
Representative dot plots for detection of IFNy+ T cell targeting vaccine-encoded Spike and non-encoded Nucleocapsid protein. Representative dot plots from intracellular cytokine staining experiments gated on CD4+ (left plots) or CD8+ T cells (right plots) are shown for one breakthrough (BTI) and one non-breakthrough (non-BTI) infection case for peripheral blood mononuclear cells restimulated Spike and Nucleocapsid peptide pool as indicated. The red square indicates the gate for defining antigen-specific IFNy+ T cells within the CD4 or CD8 T cell parent population. The frequencies of IFNy+ T cells is indicated as percent of CD4 or CD8 T cells for each shopn gate. Dot plots were produced using FlowJo version 10.

**Figure 2:**
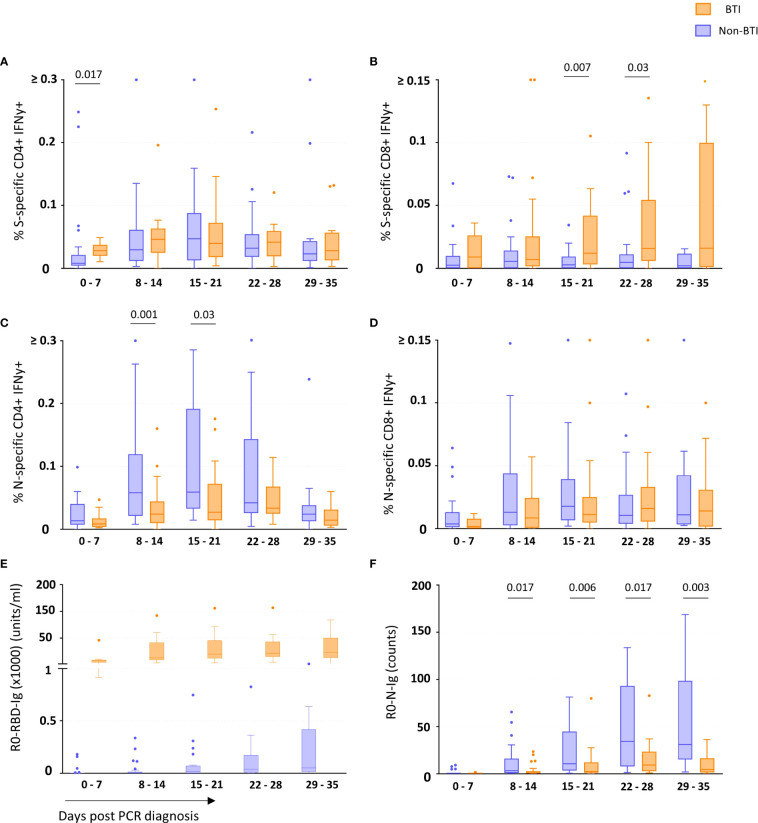
T cell and binding antibody responses targeting vaccine-encoded Spike and non-encoded Nucleocapsid protein. The frequency of Spike-specific IFNy+ CD4+ **(A)** and CD8+ **(B)** and Nucleocapsid-specific IFNy+ CD4+ **(C)** and CD8+ **(D)** T cells and concentration of binding antibodies against the Spike-Receptor Binding Domain (RBD) **(E)** and Nucleocapsid (N) **(F)** in breakthrough infections (orange boxes) and non-breakthrough infections (blue boxes) are shown as median and quartiles enclosing 50% of the datapoints, whiskers extend up to the last point inside 1.5*(IQ3 - IQ1) range (Tukey definition). Specific T cell responses were detected after *in vitro* antigen-stimulation of freshly isolated PBMCs, while the specific antibody concentrations (Spike-RBD) or counts (Nucleocapsid) (Y-axis) consider all Ig isotypes and were determined using the Roche Elecsys test. The days since first diagnosis by PCR are indicated on the x-axis. Statistical comparisons between the groups were performed using the Mann-Whitney t test. p-values below 0.05 are indicated for figure 2 **(A–F)** Spike-RBD binding Ig concentrations significantly differed between the groups during all 5 weeks post infection.

CD4 and CD8 T cell responses against the vaccine-encoded Spike (S) and the non-encoded Nucleocapsid (N) protein were studied throughout the first 5 weeks after diagnosis. The number of subject visits included within each timepoint is shown in **Table 2**. Representative dot plots and gating analyses for one BTI and non-BTI for longitudinal assessment of S- and N-specific IFNy+ CD4 and CD8 T cell frequencies after the SARS-CoV-2 diagnosis are shown in **Figure 1**. For both S- and N-specific IFNy+ CD4 T cell responses, a higher median frequency was observed (Range: 0.01 – 0.06%) as compared to the CD8 T cells (Range: 0.002 – 0.02%) (p < 0.0001, results not shown) regardless of timepoint or group affiliation. Within the S-specific T cell response, CD8/CD4 ratios were similar between BTI and non-BTI within the first two weeks of infection (0.2 - 0.4), while median CD8/CD4 ratios were 2- to 5-fold higher in BTI versus non-BTI versus during weeks 3 (p=0.06), 4 (p=0.06), and 5 (p=0.18). Within the first week of PCR diagnosis, IFNy+ S-specific CD4 T cell frequencies were significantly higher in BTI, as compared to non-BTI (p = 0.017, **Figure 2A**). Median IFNy+ S-specific CD4 T cell frequencies peaked in the second week for BTI and third week for non-BTI, consistent with an accelerated T cell response against the vaccine-encoded S upon BTI by boosting a pre-existing amnestic memory S-specific CD4 T cells. Median S-specific CD8 IFNy+ T cell frequencies in BTI gradually increased throughout the observation period of 5 weeks after PCR diagnosis but oscillated at low levels for non-BTI (**Figure 2B**) with significant differences between these groups in weeks three (p = 0.007) and four (p = 0.03) after diagnosis of SARS-CoV-2 infection. Considering the whole observation period of 5 weeks, the overall S-specific CD8 T cell response – defined as the area under the curve - was significantly higher in previously vaccinated BTI cases (AUC per day: p=0.006, results not shown). Together, these data show a positive effect of previous vaccination on IFNy+ S-specific T cell dynamics after breakthrough infection, with higher frequencies of circulating S-specific CD4 T cells early after PCR diagnosis and higher circulating S-specific CD8 T cell frequencies, when most subjects had cleared the virus from the upper airways.

**Table 2:** Subject visits included at each timepoint.

	Subject Visits (n)				
Timepoint	0 – 7	8 – 14	15 – 21	22 – 28	29 – 35
Spike and Nucleocapsid (BTI/non-BTI)^a^	11/23	25/27	22/21	18/22	12/13

a BTI, breakthrough infection; non-BTI, non-breakthrough infection.

Contrary to the S-specific CD4 T cell frequencies, N-specific CD4 T cell frequencies were lower in BTI compared to non-BTI (**Figure 2C**); considering the whole observation period, lower N-specific CD4 T cell frequencies were observed in BTI versus non-BTI group (AUC per day: p=0.042). These differences were most pronounced and significant during week 2 (p = 0.001) and week 3 (p = 0.03) after PCR diagnosis. IFNy+ N-specific CD8 T cell responses followed a similar pattern to N-specific CD4 T cell response with a trend for reduced frequencies in BTI cases over time (**Figure 2D**). However, these differences did not reach significance at any one timepoint.

### Early dynamics of antibody responses targeting the vaccine-encoded Spike and the non-encoded Nucleocapsid

We also studied whether the SARS-CoV-2 antibody response followed a similar pattern (**Figures 2** **E, F**). During BTI, S-RBD-specific antibody responses were detectable in all subjects within the first week after diagnosis and reached median levels of 7160 units/ml, 12400 units/ml, 19700 units/ml, 21350 units/ml and 23289 units/ml during weeks 1-5, respectively (**Figure 2E**). In contrast, only 6/23 of the non-BTI control subjects had detectable S-RBD-specific responses during the first week after PCR diagnosis (median level was 0.02 units/ml), while during the 2^nd^, 3^rd^, 4^th^ and 5^th^ week, median S-RBD-specific antibody levels were much lower with 5.5 units/ml, 19 units/ml, 37.9 units/ml and 50 units/ml respectively (p<0.0001 for all time points).

Similar to the “attenuated” N-specific T cell response during BTI, N-specific antibody levels were also significantly lower in BTI throughout most of the observation period (**Figure 2F**). In the first week, N-specific antibody responses were mostly undetectable in vaccinated (10/11 subject visits, median 0.13 counts) and non-vaccinated subjects (19/23 subject visits, median 0.09 counts). Thereafter the dynamics significantly differed between these groups. While the non-BTIs showed a dynamic increase of N-specific antibody responses until week 4 (median: 34 counts), antibody levels remained at significantly lower levels after BTI during following weeks with a peak at 9.2 counts in week 4 (p<0.05 for time points after week 1).

## Discussion

In our study, vaccine breakthrough infection was linked to a more rapid, earlier peaking and more extensive expansion of Spike-specific T cells and, similarly, to dramatically higher antibody levels against the vaccine-encoded S-protein compared to non-BTI. Other studies have shown similar results in the antibody response against S anti-RBD, where BTI had a higher antibody titer against the S-protein (8, 13, 14). It is noteworthy that particularly S-specific CD8+ T cell responses were superior in BTI, suggesting that “priming” with the current COVID-19 vaccines had enhanced cytotoxic CD8 T cell responses and memory cell formation. This is an interesting observation, as little is known on how to improve induction of virus-specific CD8 T cell responses by vaccination. Enhanced dynamics and acceleration of S-specific adaptive T cell responses may contribute to attenuated COVID-19 disease course, which is supported by a recent non-human primate study showing more then 10-fold higher peak airway viral loads in vaccinated animals in which CD8+ cells were experimentally depleted before exposure to SARS-CoV-2 delta variant (12). However, significantly attenuated CD4 T cell and antibody responses targeting the non-vaccine encoded N-protein were also found in BTI; attenuation of T cell and antibody responses targeting the non-vaccine encoded N antigen probably reflects reduced systemic virus dissemination and reduced *in vivo* viral loads during BTI and hence decreased stimulation of N-specific BCR/TCR, which is consistent with the attenuated disease severity upon BTI and limited inflammation and tissue dissemination in the lower respiratory tract observed in vaccinated NHP (11, 22). Reduced induction of N-specific T cells during BTI may also have a negative effect on upper airway virus control; a recent study by our group links frequencies of circulating N-specific T cells, which express IFNy upon *in vitro* peptide restimulation to early upper airway virus control and reduced systemic inflammation before seroconversion (Eser et al., 2022, manuscript submitted). Consistent with a protective role of N-specific T cells, these responses were also linked to reduced plasma concentrations of CXCL10 - a marker for COVID-19 disease severity (23). Our study has several limitations. The use of 15mer peptide pools and not taking into consideration T cell activation induced markers such as CD154 or CD137 likely underestimates the frequency of total N- and S-specific CD8 and CD4 T cells. Here, we focused on IFNy as a marker of antigen-specificity because IFNy expressing virus-specific T cells correlated with virus control for several viruses including HIV, Influenza and SARS-CoV-2 (24–27) and mechanisms of SARS-CoV-1/2 control in the upper airways by IFNy expressing N-specific T cells are being elucidated both in clinical and preclinical studies (27–29). In summary, our data show that before the emergence of Omicron variants, SARS-CoV-2 breakthrough infections are linked to enhance T cell and antibody responses targeting the vaccine encoded S-protein already early after diagnosis and to attenuate adaptive immune responses targeting the non-vaccine-encoded N-protein.

## Members of the Koco19 and CoVaKo study group

Emad Alamoudi, Jared Anderson, Maximilian Baumann, Marieke Behlen, Jessica Beyerl, Rebecca Böhnlein, Anna Brauer, Vera Britz, Jan Bruger, Friedrich Caroli, Lorenzo Contento, Jana Diekmannshemke, Anna Do, Gerhard Dobler, Ute Eberle, Judith Eckstein, Jonathan Frese, Felix Forster, Turid Frahnow, Günter Fröschl, Otto Geisenberger, Kristina Gillig, Arlett Heiber, Christian Hinske, Janna Hoefflin, Tim Hofberger, Michael Höfinger, Larissa Hofmann, Sacha Horn, Kristina Huber, Christian Janke, Ursula Kappl, Charlotte Kiani, Arne Kroidl, Michael Laxy, Reiner Leidl, Felix Lindner, Rebecca Mayrhofer, Anna-Maria Mekota, Hannah Müller, Dafni Metaxa, Leonie Pattard, Michel Pletschette, Stephan Prückner, Konstantin Pusl, Elba Raimúndez, Camila Rothe, Nicole Schäfer, Paul Schandelmaier, Lara Schneider, Sophie Schultz, Mirjam Schunk, Lars Schwettmann, Heidi Seibold, Peter Sothmann, Paul Stapor, Fabian Theis, Verena Thiel, Sophie Thiesbrummel, Niklas Thur, Julia Waibel, Claudia Wallrauch, Simon Winter, Julia Wolff, Pia Wullinger, Houda Yaqine, Sabine Zange, Eleftheria Zeggini, Thomas Zimmermann, Anna Zielke, Mohamed Ibraheem Mohamed Ahmed, Marc Becker, Paulina Diepers, Yannik Schälte, Mercè Garí, Peter Pütz, Michael Pritsch, Volker Fingerle, Ronan Le Gleut, Leonard Gilberg, Isabel Brand, Max Diefenbach, Tabea Eser, Franz Weinauer, Silke Martin, Ernst-Markus Quenzel, Jürgen Durner, Philipp Girl, Katharina Müller, Katja Radon, Christiane Fuchs

Members of the CoVaKo study group: Prof. Dr. Helmut Messmann, Dr. Andre Fuchs, Dr. Alanna Ebigbo, Dr. Christoph Römmele, Maximilian Ullrich, Marie Freitag, Prof. Dr. Claudia Traidl-Hoffmann, Mehmet Goekkaya, Aline Metz, Corinna Holetschek, Prof. Avidan Neumann, Elisabeth Kling, Prof. Dr. Reinhard Hoffmann, Mihail Pruteanu, PD Dr. Thomas Wibmer, Dr. Susanne Rost, Prof. Dr. Klaus Überla, Dr. Philipp Steininger, Monika Wytopil, Stephanie Beileke, Dr. Sandra Müller-Schmucker, Tamara Hastreiter, Kirsten Fraedrich, Dr. Klaus Korn, Dr. Frank Neumann, Dr. Claudia Kuhn, Dr. Katja Günther, Dr. Elke Friedrich, Prof. Dr. Michael Hoelscher, PD Dr. Andreas Wieser, PD Dr. Christof Geldmacher, Christian Janke, Michael Plank, Jessica Guggenbühl, Christina Reinkemeyer, Ivan Noreña, Dr. Noemi Castelletti, Dr. Raquel Rubio Acero, Dr. Mohamed Ibraheem Mahmoud Ahmed, Paulina Diepers, Tabea M. Eser, Anna Fuchs, Olga Baranov, Bernadette Bauer, Danni Wang, Ivana Paunovic, Prof. Dr. Ulrike Protzer, Samuel Jeske, Catharina Christa, Kathrin Tinnefeld, Martin Vu, Annika Willmann, Dr. Hedwig Roggendorf, Dr. Nina Körber, Dr. Tanja Bauer, PD Dr. Sabine Gleich, Prof. Dr. Ralf Wagner, Dr. Claudia Asam, Sebastian Einhauser, Manuela Weps, Antonia Ebner, Maria José de Schultz, Cedric Rajes, Aya Al Wafai, David Brenner, David Brenner, Laura Sicheneder, Melanie Berr, Anja Schütz, Dr. Stilla Bauernfeind, Prof. Dr. Dr. André Gessner, Prof. Dr. Barbara Schmidt, Daniela Biermeier, Dr. Benedikt Lampl, Ulrich Rothe, Dr. Ute Gleißner, Dr. Susanne Brückner, Michaela Treml, Holger Schedl, Dr. Beate Biermaier, Markus Achatz, Dr. Daniela Hierhammer, Johanna Englhardt, Werner Scheidl, Dr. Sivaji Jeyaraman, Dr. Barbara Schutt, Prof. Dr. Johannes Liese, Prof. Dr. Martina Prelog, PD Dr. Giovanni Almanzar, Valeria Schwägerl, Dr. Julia Bley, Tim Vogt, Kimia Kousha, Lars Ziegler, Astrid Stein, Dr. med. Johann Löw, Barbara Finkenberg, Dennis Pollak, Alexander Zamzow, Dr. Nicole Eberbach, Lara Balkie, Tanja Kretzschmann, Matthias Gehrig, Matthias Bandorf, Kilian Keck, Dr. Jan Allmanritter, Shahid Rafique, Mona Finster, Dr. med. Ingo Baumgart, Sabine Heumüller-Klug, Hans-Jürgen Koglin, Prof. Dr. Olaf Gefeller, Dr. Christine Gall, Prof. Dr. Annette B. Pfahlberg, Isabelle Kaiser, Prof. Dr. Jörg Scheidt, Johannes Drescher, Yannic Siebenhaar, Dr. Florian Wogenstein, Dr. Dirk Reinel, Prof. Dr. Beatrix Weber, Fabian Zarzitzky, Prof. Bernhard Liebl, Prof. Caroline Herr, Dr. Katharina Katz, Prof. Andreas Sing, Dr. Alexandra Dangel

## Data availability statement

The raw data supporting the conclusions of this article will be made available by the authors, without undue reservation.

## Ethics statement

The studies involving human participants were reviewed and approved by Ludwig Maximilian University of Munich, Germany. The patients/participants provided their written informed consent to participate in this study.

## Author contributions

MA, PD, TE, BB, AF, and DW contributed to the experimental work and gating analyses. MA, NC, and OB performed the statistical analysis and prepared figures. The human cohort studies were conceived and planned by KÜ, JL, PS (CoVaKo), CG, IK, AW, LO, and MH (KoCo19-sub-study). CG conceived the immunological study presented here. CJ, MiP, IK, and CR conducted the clinical work. All authors contributed to study data discussion, writing and revision of the manuscript.
